# Validation of the Spanish version of the Oxford knee score and assessment of its utility to characterize quality of life of patients suffering from knee osteoarthritis: a multicentric study

**DOI:** 10.1186/s12955-017-0761-2

**Published:** 2017-09-29

**Authors:** Jesús Martín-Fernández, Roberto García-Maroto, Fco Javier Sánchez-Jiménez, Alonso Bau-González, Homero Valencia-García, Blanca Gutiérrez-Teira, Juan Carlos Arenaza, Lidia García-Pérez, Renata Linertová, Amaia Bilbao

**Affiliations:** 1 C° Villamanta (C.S. Navalcarnero). Gerencia Asistencial de Atención Primaria. Servicio Madrileño de Salud, Avda Libertad 21 s/n, Villamanta, 28610 Madrid, Spain; 2 Facultad de Ciencias de la Salud. Universidad Rey Juan Carlos, Avda Atenas s/n, 28922 Alcorcón, Madrid, Spain; 3 Red de Investigación en Servicios de Salud en Enfermedades Crónicas, Bilbao, Spain; 4 Servicio de Traumatología. Hospital Universitario Clínico San Carlos. Servicio Madrileño de Salud, C/ Profesor Martín Lagos, S/N, 28040 Madrid, Spain; 5 C.S. Gregorio Marañón. Gerencia Asistencial de Atención Primaria. Servicio Madrileño de Salud, Calle Polvoranca, 65. 28923 Alcorcón, Madrid, Spain; 6 Servicio de Traumatología. Hospital Universitario Rey Juan Carlos, C/ Gladiolo s/n, 28933 Móstoles, Madrid Spain; 7 Servicio de Traumatología. Hospital Universitario Fundación Alcorcón, C/ Budapest, 1 28922 – Alcorcón, Madrid, Spain; 8 C. S. El Soto. Gerencia Asistencial de Atención Primaria. Servicio Madrileño de Salud, Avenida Olímpica, 38, 28935 Móstoles, (Bizkaia) Spain; 9 Servicio de Traumatología y Cirugía Ortopédica, Hospital Universitario Basurto (Osakidetza), Avda. Montevideo, 18, 48013 Bilbao, (Bizkaia) Spain; 10 Fundación Canaria de Investigación Sanitaria (FUNCANIS), Camino Candelaria N° 44, 1ª planta, 38109 El Rosario (Santa Cruz de Tenerife), Spain; 11 Unidad de Investigación, Hospital Universitario Basurto (Osakidetza), Avda. Montevideo, 18, 48013 Bilbao, (Bizkaia) Spain

**Keywords:** Quality of life, Health status, Osteoarthritis, knee, Validation studies

## Abstract

**Background:**

Knee osteoarthritis (OA) represents a heavy burden for patients and the society as a whole. The Oxford Knee Score (OKS) is a well known tool to assess the quality of life in patients with Knee OA. The purpose of this study was to analyze the psychometric properties of the Spanish version of the OKS, including its reliability, validity, and responsiveness.

**Methods:**

Prospective observational study that included 397 patients diagnosed with knee OA according to the criterion of the American Rheumatism Association, which were recruited in 3 different Spanish regions. Their self-perceived health-related quality of life (HRQL) was assessed through 3 questionnaires: a generic one (the EQ-5D-5 L) and two specific ones adapted to Spanish (the Western Ontario and McMaster Universities Osteoarthritis Index (WOMAC) and the Oxford Knee Score (OKS). The follow-up period was 6 months, and the acceptability of the OKS was evaluated, together with its psychometric properties, presence of ceiling and floor effects, validity, reliability, and sensitivity to change.

**Results:**

The OKS was fully answered in 99.5% of cases, with no evidence of ceiling or floor effects. Its factor structure can be explained in a single dimension. Its discriminating capacity was very good compared to the groups generated by the WOMAC and the EQ-5D-5 L. The correlation of the OKS with the dimensions of the latter questionnaires was around 0.7. The test-retest reliability was excellent (ICC 0.993; CI 95%: 0.990–0.995) and so was its internal consistency (Cronbach’s α = 0.920). The effect size was 0.7 for moderate improvements in the HQRL, which is similar to that of the dimensions of the WOMAC and greater than for the EQ-5D-5 L. The minimum clinically significant difference that was detected by the questionnaire was 6.1 points, and the minimum detectable change was 4.4 points.

**Conclusions:**

The Spanish-adapted version of the OKS is a useful, valid tool for assessing the perceived HRQL in patients suffering from knee OA, with psychometric properties similar to the WOMAC, and that allows for discriminating the patient’s condition at a particular moment as well as for appraising changes over time.

**Electronic supplementary material:**

The online version of this article (10.1186/s12955-017-0761-2) contains supplementary material, which is available to authorized users.

## Background

Osteoarthritis (OA) is the most frequent joint disease, characterized by progressive articular cartilage loss that results in joint pain and functional impairment, which impacts the ability to perform daily-life activities. The prevalence of this type of disease is very high affecting 4% of the general population worldwide based on radiological diagnosis, and up to 20% in the case of specific population groups, such as women over 60 years [1]. Knee OA is a heavy burden for patients and the society as a whole. International studies have estimated that knee and hip OA constituted 0.7% of all disability adjusted life years (DALY) lost in 2010, a 40% increase with respect to 1990 [1]. Eighty-three percent of DALY lost due to OA are due to OA of the knee [2].

Knee OA entails a substantial impact on health related quality of life (HRQL) [3, 4]. HRQL is generally considered to incorporate the evaluation of functioning status as well as the patient’s perception of their emotional functioning and social role. Since patients’ responses vary greatly in the face of identical stressors, such as pain, HRQL is a crucial outcome measure [5]. The dimensions of HRQL most affected by knee OA are those related to physical activity and self-efficiency [6]. It seems that knee OA has a greater impact on the physical aspects of HRQL in the case of women, whereas men report worse scores on psychological-related scales [7]. Besides, HRQL predicts future inpatient and outpatient health care utilization and mortality in patients diagnosed of OA [8]. Therefore, measures of HRQL are important not only for assessing the burden of the disease or the results of any intervention, but also for helping informed decision-making in the allocation of often limited health resources [4].

In the case of knee OA, there are several specific tools to measure HRQL, some of which have been adapted and validated for the Spanish setting, such as the Western Ontario and McMaster Universities Osteoarthritis Index (WOMAC) –a useful questionnaire for the assessment of OA of the lower limb [9, 10]–, the “Knee Society Clinical Rating System” (KSS) [11, 12], or the Knee Injury and Osteoarthritis Outcome Score (KOOS) [13]. Other questionnaires like the “Oxford Knee Score” (OKS) have not had their psychometric properties validated for our setting. The OKS is a brief, 12-item, self-reported scale developed to measure the impact of total knee replacement surgery on the perception of HRQL by patients [14], and its scores and outcome interpretations have been slightly modified throughout the years it has been in use [15]. It is reported to be amongst the most sensitive, responsive, reliable, and valid patient-reported questionnaire for knee conditions [16]. It has been adapted and validated into Italian [17], Dutch [18], Chinese and Singapore English [19], German [20], French [21], Japanese [22], Portuguese [23], Korean [24], Persian [25], Greek [26], Spanish in Colombia [27], Arabic language [28], and Finnish language [29]. Owing to its good psychometric properties, it has been favorably compared to other widely used tools in different languages that are more difficult to administer [30, 31]. Although the OKS has been adapted to Spanish for Spain, its psychometric properties have not been assessed in the Spanish population setting. As far as we know, only the Dutch and Finnish language adaptations of OKS have been validated in a prospective manner, similarly to the original work by Dawson et al. [18, 29], whereas its factor structure has not been confirmed in any of its adapted versions.

In Spain, knee OA implies an enormous burden of illness for the people who suffer from it and for the whole society [32], and is therefore worthy of being measured. There are new instruments that serve this purpose, such as the OKS; however, for a questionnaire to be useful in culturally different areas with different languages, it must not only be translated into the new language but also adapted to account for any different or new cultural characteristics. The adaption must then be validated as the original version was. This work tackles the study of the psychometric properties of the OKS in its Spanish-adapted version, including its reliability, validity, and responsiveness.

## Methods

### Design

Prospective observational study. A population sample was recruited and followed up after 6 months.

### Sampling and sample size

Opportunistic sampling of patients diagnosed with knee OA was performed both in traumatology and primary care consultations in Bizkaia, Madrid, and Tenerife. Patients were included in a consecutive way between January and December 2015. All patients were chronic, and the knee OA was diagnosed according to the American Rheumatism Association’s criterion [33], either by the clinician that included the patient in the study or from what was already recorded in the clinical history. Patients with OA from other regions and those suffering other comorbidities were also included. Subjects that did not properly understand or read Spanish and those diagnosed with any cognitive impairment were excluded.

The confirmatory factor analysis (CFA) set the minimum requirements to calculate sample size since it was the most stringent of the employed analytic methods in this regard. It was estimated that 300 patients would be needed, using a questionnaire with a single factor comprised of 12 items [34]. This sample size would also allow for estimating intraclass correlation coefficients (ICC) of >0.8 with precision values <10% [35].

All included patients provided written consent for participation and the study was approved by the relevant Ethics Committees for Clinical Research.

### Variables

The personal variables recorded for each patient were age, gender, body mass index (BMI), joints affected by arthritis, previous joint replacement surgeries, and Charlson’s index, which was calculated to assess comorbidity situations [36]. Patients answered three questionnaires, all in their Spanish version, in order to appraise their HRQL: a generic one (EQ-5D-5 L) [37], and two specific to OA (the WOMAC [9] and the OKS [14]).

The EQ-5D-5 L Spanish for Spain version has shown initial content and face validity [37]. This new version improves the old EQ-5D-3 L version, which had high internal consistency and reliability levels but, on the contrary, showed ceiling effect and low responsiveness [38].The EQ-5D-5 L asks about current self-perception of health and is comprised of two parts. The first part includes 5 questions on general health: mobility, self-care, performance of daily-life activities, pain/discomfort, and anxiety/depression. Each dimension is measured on a scale from 1 to 5. A single weighted score for health condition is then obtained from these 5 questions, the so-called utility index, and the higher the score the better the health status [39]. The second part consists of a visual analogue scale (VAS) that ranges from 0 (worst health condition) to 100 (best health condition).

The WOMAC [9] is a self-administered questionnaire, specific to patients suffering from OA of the hip or knee. It has a multidimensional scale comprised of 24 items clustered according to 3 domains: pain (5 items), stiffness (2 items), and physical functionality (17 items). Its Likert version, where each item receives a score from 0 to 4 corresponding to the different intensity levels of the response (none, light, moderate, severe, extreme), was chosen. This score is summed and standardized from 0 (best ability) to 100 (worst ability). The greater the score, the worse the health condition of the patient. This questionnaire has been adapted and validated for our setting. The adapted version of the WOMAC questionnaire showed high convergent validity, internal consistency (Cronbach’s α ranging from 0.81 to 0.93), and test-retest reliability. The responsiveness test showed effect sizes ranging from 1.5 to 2.2 in patients that had undergone hip replacement [10].

The OKS is a self-administered questionnaire that can be answered via “face to face” interviews or mailed-in by the patient after completion. It contains 12 questions, with 5 possible answers each, intended to evaluate the patient’s perception of quality of life over the last 4 weeks. It has been used both to assess the baseline situation and to study changes after prosthetic implants in patients suffering from knee OA. Each answer is given a score from 0 to 4, where 4 is the best possible result [15]. After being summed up, a total score is obtained that ranges from 0 to 48, where 48 is the best possible outcome. The Spanish-adapted version was created under agreement with the Oxford University Innovation™, following a process of translation and inverse retro-translation (Additional file 1).

Subjects recruited in Madrid were interviewed 7 to 15 days after the inclusion visit, and the OKS questionnaire was repeated after ensuring that there were no changes in their health condition. All included patients were interviewed again after a follow-up period of 6 months: they were asked if they had undergone replacement surgery, the EQ-5D-5 L, WOMAC, and OKS questionnaires were repeated, and transition questions were posed to assess if their general health self-perception had suffered any changes.

### Statistical analysis

Continuous variables were described by their measures of central tendency and dispersion, whereas discrete variables were described by their percentages. Confidence intervals were set at 95%.

#### Acceptability and floor and ceiling effects

The number of unfilled questionnaires and unanswered questions was noted.

Ceiling or floor effects were considered to be present if more than 15% of respondents reported the highest or lowest possible score, respectively [40].

#### Analysis of the psychometric properties

#### Validity

The validity of the construct was appraised via an explanatory factor analysis (EFA) that analyses the unidimensionality of the questionnaire. The sampling adequacy was checked using Barlett’s test of sphericity and Kaiser-Meyer-Olkin (KMO) test. The null hypothesis of Barlett’s test is that the correlations matrix is a singular matrix. The rejection of this hypothesis allows for confirming the existence of linear relationships between the factors and the explained variable. The KMO sampling adequacy test is a measure of the covariance among variables and values >0.90 are considered to be optimal [41]. Both factor loading (values >0.40 were considered optimal) and commonalities were noted, which together account for the percentage of the item’s variance explained by each factor.

To complement our results, a CFA for categorical variables was also performed. The robust unweighted least squares estimator was used and several fit indices were calculated [42, 43]: the root mean square error of approximation (RMSEA), for which a value <0.08 was considered acceptable, and the Tucker-Lewis Index (TLI) and Comparative Fit Index (CFI), both of which had to be >0.95 to be considered satisfactory [44]. Factor loadings were also examined and those ≥0.40 were considered acceptable. Therefore, the model was considered adequate when these acceptability criteria were met.

The scores obtained through the OKS were compared to the terciles of the distributions obtained from the EQ-5D-5 L and WOMAC questionnaires in order to assess the validity of the known groups.

Convergent validity was checked through the correlations of the OKS scale with the WOMAC and EQ-5D-5 L (utility index and VAS) scales. Pearson’s r or Spearman’s rho were used to study such correlations, and 0.7 was set as the threshold for considering strong associations to be present [40].

#### Reliability

Internal consistency was tested using Cronbach’s α [45] that was obtained from the scores of the inclusion visit. This coefficient summarizes internal correlations of all the elements of a scale. The greater the coefficient (range 0.0–1.0), the greater the internal consistency of the scale and the greater the probability for a single dimension to be underlying the questionnaire. For a single-dimension tool comprising 12 components, Cronbach’s α is expected to reach values >0.85 in order for its internal consistency to be considered optimal [46].

The test-retest reliability was studied in the sub-sample from Madrid, and ICCs were used to compare the test against the retest scores. According to the classification proposed by other reliability measures [47], ICC values >0.7 are considered acceptable and >0.9 optimal.

#### Responsiveness

The OKS questionnaire was repeated at a follow-up period of 6 months to evaluate its responsiveness to changes resulting from disease progression. In order to assess changes in the knee condition compared to the 6 previous months as perceived by patients, transition questions were posed and answered on a scale comprising 5 answers (much worse, slightly worse, same, slightly better, or much better than before). These questions were aimed at appraising the sensitivity of the OKS questionnaire to change. In the case of the WOMAC, transition questions were answered on the same scale, but they were specific for each of its domains (pain, stiffness, and physical functionality).

Changes were appraised with the OKS by subtracting initial from final scores, so that positive values indicate an improvement in general condition. The procedure was the same with the EQ-5D-5 L but, in the case of the WOMAC, the final scores were subtracted from the initial ones so that positive values also indicated improvements. Transition questions were posed to every group of patients in order to see if significant changes had occurred, and basal scores were contrasted against those at 6 months of follow-up. The relationship between the median and standard deviation was calculated to determine the effect size of the change for each group of patients: values >0.5 were regarded as moderate change, and values >0.8 as large change [48]. The effect size was then compared to the one obtained from the WOMAC and EQ-5D-5 L scales.

Furthermore, the minimal clinically important difference (MCID) and the minimal detectable change (MDC) were determined. These two measures are related to responsiveness, but are more clinically oriented and focused at the individual level. Average change in patients that had experienced moderate improvement in their condition (reported feeling “slightly better”) was used to calculate MCID at the 6 months follow-up [49].

The MDC expresses the minimal magnitude of change above which the observed change is likely to be real and not just measurement error. For estimation of MDC, the standard error of measurement (SEM) was determined, which quantifies the precision of individual scores on a test. The SEM was estimated as the square root of the mean square error term from the ANOVA [50, 51]. From the SEM, the MDC was derived as follows [40, 50]: $MDC= SEM\times z- score\times \sqrt{2}$. A 95% confidence level (MDC_95%_) was set, corresponding to a z-value of 1.96. The interpretation of MDC_95%_ is that if a patient shows a score change equal to or greater than the MDC_95%_ threshold, it is possible to state with 95% confidence that this change is reliable and not the result of a measurement error. Finally, the MCID was divided by the MDC_95%_ to determine if the MCID surpassed the MDC_95%_ [52]: if this ratio exceeded 1, the MCID could be discriminated from measurement error.

All effects were considered statistically significant at *p* < 0.05. The statistical analyses were performed using SPSS 18.0 and Mplus 6.1 software.

## Results

A total of 397 patients were included: 158 in Bizkaia, 158 in Madrid, and 81 in Tenerife. Of them, 36.8% were recruited at primary care, 55.2% at traumatology, and 8.0% at rheumatology consultations. The mean time elapsed since diagnosis was 61.6 months (CI 95%: 55.6–67.6 months). Women comprised 69.8% (CI 95%: 65.3–74.3%) of the sample, with an average age of 71.4 years (CI 95%: 70.5–72.3 years).

In terms of the knee affected by OA, in 27.7% (CI 95%: 23.3–32.1%) of cases it was the right knee, in 30.0% (CI 95%: 25.5–34.5%) the left knee, and in 43.3% (CI 95%: 38.4–48.2%) both knees.

Total knee replacement surgery had been previously performed in 18.1% (CI 95%: 14.3–21.9%) of cases. The average Charlson’s comorbidity index was 0.8 points (CI 95%: 0.7–0.9), and mean BMI was 29.7 (IC 95%: 29.2–30.2).

Table 1 shows the outcome from the responses given by patients to the OKS, WOMAC, and EQ-5D-5 L questionnaires.

**Table 1:** Summary of the outcome from the OKS, WOMAC, and EQ-5D-5 L questionnaires

	n	Average score (IC 95%)	Median (Interquartile range)
OKS	395	22.0 (21.0–22.9)	20.0 (14.0–27.0)
WOMAC pain	396	46.2 (44.1–48.3)	44.4 (30.0–60.0)
WOMAC rigidity	396	45.5 (42.9–48.0)	46.4 (25.0–62.5)
WOMAC impairment	397	52.7 (50.5–55.0)	48.5 (25.0–62.5)
EQ-5D-5 L utility	393	0.54 (0.52–0.57)	0.62 (0.39–0.74)
EQ-5D-5 L VAS	390	57.0 (55.2–59.5)	60.0 (45.0- 75.0)

*OKS* Oxford Knee Score. Range 1–48. The better the score, the better the health condition

*WOMAC* Western Ontario and McMaster Universities Osteoarthritis Index. Scale range 1–100. The better the score, the worse the health condition

EQ-5D-5 L Utility score range 0–1, where 0 = condition comparable to death, and 1 = perfect health, although negative scores are allowed

EQ-5D-5 L VAS: Visual Analogue Scale. Range 0–100, where 0 = worst health condition, and 100 = best conceivable health condition

### Acceptability and floor and ceiling effects

Information was obtained in 395 cases (99.5%; CI 95%: 98.8–100%) which allowed summarizing the results from the OKS questionnaire. Questions 7, 9, and 12 were answered in all cases, and questions 1, 2, 3, 5, 6, 10, and 11 in all cases but one. Question 8 was not answered in 2 occasions, and question 4 in 6 cases. All possible answers, namely all ranges of response (0 to 4), were posed for every question. Only in the case of question 7, 48% of responses were concentrated in the top score. Only in the case of questions 1 and 7, less than 10% of responses clustered into the bottom end of the scale (0 and 1), which did not happen for the top end in any case. For the total score, there was no aggregation at the low end of the scale and only 0.25% and 0.61% of the responses scored 48 out of 48 possible points in the inclusion visit or in the six month visit respectively. Hence, the presence of floor or ceiling effects was ruled out.

### Validity

With regards to the validity of the construct, a unidimensional structure was found in the EFA with a single factor that explained 55.5% of variance (KMO = 0.946, Bartlett’s test of sphericity χ^2^ = 2597, 66 degrees of freedom, *p* < 0.001). All factor loadings were >0.50, and commonalities were >0.40 except for questions 4 and 8 (Table 2).

**Table 2:** Exploratory factor analysis of items in the Oxford Knee Score (OKS)

	EFA 1 factor	
Variable	Factor loading	Commonality
OKS 1	0.761	0.579
OKS 2	0.751	0.564
OKS 3	0.774	0.599
OKS 4	0.572	0.327
OKS 5	0.796	0.634
OKS 6	0.773	0.597
OKS 7	0.670	0.449
OKS 8	0.605	0.365
OKS 9	0.839	0.705
OKS 10	0.767	0.589
OKS 11	0.788	0.620
OKS 12	0.794	0.630

*EFA* Exploratory factor analysis

*OKS* Oxford Knee Score

**Fig. 1:**
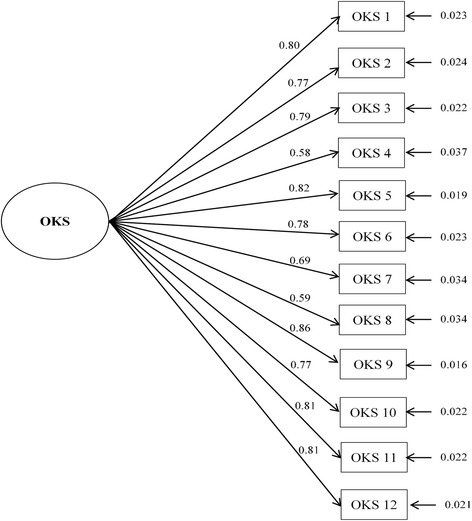
Confirmatory factor analysis for categorical data of the Oxford Knee Score (OKS) questionnaire. Standardized parameters and standard errors are shown. Fit indices are as follows: χ^2^ = 175.40, degrees of freedom =54, *p* < 0.0001; RMSEA (CI 90%) =0.076 (0.064–0.089); CFI =0.981; TLI =0.977

The results of the CFA (Fig. 1) showed excellent fit indices: (a) the RMSEA was 0.076, that is <0.08; and (b) the CFI and TLI were 0.981 and 0.977, respectively, exceeding the benchmark of 0.95. Factor loadings were all statistically significant (*P* < 0.001), ranging from 0.58 to 0.86 (Fig. 1).

The validity of known groups, which measures the discriminatory capacity of the questionnaire, can be checked in Table 3, where mean scores and 95% CI of the OKS are shown for the various terciles of the WOMAC and EQ-5D-5 L scales distribution. Differences between the three groups are clearly observed in the OKS scores, with average changes of 5.6 and 11.9 points per tercile.

**Table 3:** Average scores of the OKS in patients after being clustered according to the terciles obtained from the WOMAC and EQ-5D-5 L questionnaires

	OKS score for the lower tercile of the questionnaire distribution (CI 95%)	OKS score for the middle tercile of the questionnaire distribution (CI 95%)	OKS score for the top tercile of the questionnaire distribution (CI 95%)
WOMAC pain	31.4 (30.0–32.7)	20.5 (19.5–21.6)	13.8 (12.7–14.9)
WOMAC rigidity	29.7 (28.2–31.3)	22.1 (20.8–23.4)	14.4 (13.3–15.4)
WOMAC impairment	32.0 (30.8–33.3)	20.1 (19.1–21.0)	13.1 (12.1–14.0)
EQ-5D-5 L utility	13.6 (12.5–14.6)	21.1 (20.0–22.2)	31.1 (29.7–32.4)
EQ-5D-5 L VAS	15.7 (14.0–17.3)	21.5 (20.3–22.7)	27.1 (25.3–28.9)

*OKS* Oxford Knee Score

*WOMAC* Western Ontario and McMaster Universities Osteoarthritis Index

*VAS* Visual Analogue Scale

Table 4 shows the correlations between the OKS scores and WOMAC domains or EQ-5D-5 L VAS and utilities. Due to the different ways in which scores are presented on the scales, negative correlations with the WOMAC and positive ones with the EQ-5D-5 L were to be expected. All associations were strong except for rigidity on the WOMAC scale, whose correlation was at the limit of the required threshold, and the EQ-5D-5 L VAS.

**Table 4:** Correlations (Pearson’s r) between the scores from the OKS, WOMAC scales, and EQ-5D-5 L scales (utility index and VAS)

	OKS	WOMAC pain	WOMAC rigidity	WOMAC impairment	EQ-5D-5 L Utility	EQ-5D-5 L VAS
OKS	_					
WOMAC pain	−0.745*	_				
WOMAC rigidity	−0.641*	0.643*	_			
WOMAC impairment	−0.849*	0.799*	0.719*	_		
EQ-5D-5 L utility	0.756*	−0.623*	−0.504*	−0.706*	_	
EQ-5D-5 L VAS	0.530*	−0.472*	−0.361*	−0.516*	0.547*	_

*p < 0,001

*OKS* Oxford Knee Score

*WOMAC* Western Ontario and McMaster Universities Osteoarthritis Index

Higher scores indicate better health condition in the OKS and EQ-5D-5 L, and opposite in the case of the WOMAC

### Reliability

Regarding internal consistency, Cronbach’s α was 0.920 for the OKS questionnaire.

The ICC for the 158 subjects that repeated the questionnaire at 7 and 14 days after the inclusion visit was 0.993 (CI 95%: 0.990–0.995) and Cronbach’s α was 0.997 at both check points.

### Responsiveness

After 6 months, follow-up was possible in the case of 331 subjects. Of those, 42 people had undergone joint replacement surgery. Thirty-three patients (10.1%; CI95%: 6.7–13.2%) received some sort of rehabilitation or physical therapy during this period. One hundred and one patients (30.5%; CI 95%: 25.6–35.5%) reported feeling “slightly better” or “much better” when asked about the knee that caused their inclusion in the study, and 143 (43.2%; CI 95%: 37.9–48.5%) stated they felt “slightly worse” or “much worse”.

Tables 5 and 6 show the average change in the scores of the diverse questionnaires employed when the patient had perceived a change in health condition. When the OKS was used, the effect size of the change was 0.69 for subjects that stated feeling “slightly better” and 1.60 if they felt “much better”. The effect size was lower for negative changes, with a value of 0.24 for moderate (“slightly worse”) changes and 0.57 in the case of substantial (“much worse”) negative changes. There was a clear gradient in the score depending on the change perceived by the patient, which is significantly different for groups that reported feeling “slightly worse”, “slightly better”, or “much better”. There is a small overlap between those who felt “much worse” and “slightly worse”. The tool proved to be more sensitive than the EQ-5D-5 L and worked in a similar way to the WOMAC scales on pain and impairment, whereas the scale on rigidity was less sensitive to change.

**Table 5:** Changes in the OKS, and EQ-5D-5 L questionnaires observed after a follow-up period of 6 months in patients that reported changes in their condition

	The knee condition is “much worse” *N* = 59		The knee condition is “slightly worse” *N* = 84		The knee condition is “slightly better” *N* = 56		The knee condition is “much better” *N* = 45	
	Average change (CI 95%)	E.S.	Average change (CI 95%)	E.S.	Average change (CI 95%)	E.S.	Average change (CI 95%)	E.S.
OKS	−4.5 (−6.6– −2.4)	0.57	−1.6 (−2.9– −0.2)	0.24	6.1 (3.7–8.5)	0.69	17.4 (14.1–20.7)	1.60
EQ-5D-5 L utility	−0.17 (−0.25– −0.08)	0.55	−0.01 (−0.06–0.03)	0.07	0.14 (0.07–0.20)	0.43	0.30 (0.21–0.39)	0.96
EQ-5D-5 L VAS	−4.6 (−12.8–3.7)	0.15	−0.74 (−4.9–3.4)	0.04	7.9 (3.0–12.8)	0.58	13.2 (9.0–17.4)	0.94

*E.S.* Effect size

*OKS* Oxford Knee Score

Basal scores were substracted from final scores, so that positive outcomes indicate improvement

**Table 6:** Changes in the WOMAC questionnaire observed after a follow-up period of 6 months in patients that reported changes in their condition

	Pain is “much worse” *N* = 51		Pain is “slightly worse” *N* = 82		Pain is “slightly better” *N* = 61		Pain is “much better” *N* = 47	
WOMAC pain	Average change (CI 95%)	E.S.	Average change (CI 95%)	E.S.	Average change (CI 95%)	E.S.	Average change (CI 95%)	E.S.
	−9.9 (−14.9– −4.8)	0.55	−2.0 (−5.2–1.3)	0.13	17.7 (13.1–22.3)	0.98	28.8 (21.5–36.1)	1.15
	Rigidity is “much worse” *N* = 44		Rigidity is “slightly worse” *N* = 85		Rigidity is “slightly better” *N* = 52		Rigidity is “much better” *N* = 42	
WOMAC rigidity	Average change (CI 95%)	E.S.	Average change (CI 95%)	E.S.	Average change (CI 95%)	E.S.	Average change (CI 95%)	E.S.
	−13.1 (−20.9– −5.3)	0.51	−0.7 (−5.9–4.5)	0.03	12.3 (5.1–19.4)	0.48	30.1 (21.9–38.2)	1.15
	Impairment is “much worse” *N* = 47		Impairment is “slightly worse” *N* = 90		Impairment is “slightly better” *N* = 47		Impairment is “much better” *N* = 31	
WOMAC impairment	Average change (CI 95%)	E.S.	Average change (CI 95%)	E.S.	Average change (CI 95%)	E.S.	Average change (CI 95%)	E.S.
	−10.5 (−15.2– −5.7)	0.65	−2.4 (−5.5–0.7)	0.16	18.4 (12.3–24.4)	0.89	38.6 (29.7–47.6)	1.58

*E.S.* Effect size

*WOMAC* Western Ontario and McMaster Universities Osteoarthritis Index

Final scores were substracted from basal scores. This way, positive outcomes indicate improvement

For subjects that experienced a “moderate” subjective improvement, the average change in the OKS was 6.1 points (SD = 8.9), which was used to estimate the MDCI. The SEM was estimated to be 1.5, so MDC_95%_ was calculated to be 4.38, which means that the ratio MDCI / MDC_95%_ was 1.4.

## Discussion

The Spanish version of the OKS questionnaire is a reliable, sensitive to changes, valid tool to measure HRQL in patients that suffer from knee OA. Given the extraordinarily high response rate, it also is a well accepted questionnaire.

The validity of the OKS was assessed from different perspectives, although its “apparent” validity has not been tested since it is an adaptation. Discriminatory or known-groups validity seems adequate since the outcome score differs greatly in subjects with very different scores on the WOMAC or the EQ-5D-5 L scales. Additionally, it does not show significant ceiling or floor effects that compromise such discriminatory capacity, as has been previously pointed out in other adaptations [17, 20, 21, 29].

The convergent validity of the tool seemed appropriate. Correlations of the OKS adapted-version with the specific scales of the WOMAC or the generic scales of the EQ-5D-5 L were stronger than those found between the original version and other generic tools that measure HRQL [14, 30]. In the case of adaptations of the OKS to other languages, like Portuguese [23] or German [20], these correlations were similar or slightly stronger. This way of measuring convergent validity offered better results for the OKS than those reported in other questionnaires like the Spanish version of the KSS [12].

Construct validity of the OKS was also studied. Its factorial structure sustains the unidimensionality of the questionnaire. In the EFA, all items were found to consistently saturate the same factor and showed higher values than the English version [14]. Acceptable values of RMSEA were obtained during the CFA, and TLI and CFI were optimal [44]. Although the possibility of disaggregating pain and impairment components from the OKS has been proposed, this unifactorial structure seems to be the most solid one [53], which is supported by this outcome.

Internal consistency was better than for the original scale in the inclusion period (Cronbach’s α =0.92 vs. 0.87) [14]. The test-retest reliability was very high and the obtained values, measured through the ICC, allowed to qualify the tool as reliable [40].

The discriminatory capacity of the questionnaire was adequate, which accounts for its ability to distinguish between individuals in different situations, but the tool can also be used to study the perception changes of a single person’s situation, which means that its evaluative capability is adequate [54]. In fact, this tool was designed for that purpose and the outcome of this study supports this type of use. The effect size of the change for “moderate” positive changes was similar to the WOMAC but slightly lower than the set benchmark of 0.8. In the validation assessment of the original version, the observed effect size of the change after surgery was 2.1 [14], which is only comparable to really substantial improvements (effect size =1.6) since it was tested in patients that had undergone knee replacement. The evaluative capacity was greater to detect positive than negative changes, as is the case with other questionnaires [55], although even in the case of negative changes the capacity of this tool is similar or higher than the WOMAC, and greater than the generic EQ-5D-5 L questionnaire.

The MCID was 6.1 in the case of subjects that had experienced moderate improvement. Values of MCID between 3 and 5 points had been proposed for the validation of the original version [15] and confirmed in subsequent studies [56], although these studies only included subjects that had undergone knee replacement surgery. The MCD_95%_ was 4.38, which represents the lowest score change (at the particular patient level) that is not the result of measurement error of the instrument, and can be understood as the lowest bound of real change, although it may not indicate clinical significance [50]. The ratio between the MCID and MDC_95%_ was higher than 1, indicating that the MCID can be discriminated clearly from measurement error.

This work has some limitations. The studied subjects may not be representative of the national population. Patients were included from different regions and at different stages of the disease evolution, although we did not record or classify the knee OA severity of each patient. Besides, there are intrinsic limitations to the methodology used to assess the psychometric characteristics (the classical test theory), with its assumptions and weaknesses, but the validation process has been complemented with a CFA specific for categorical data that scrutinizes such deductive assumptions using statistical analysis [42].

The outcome of this study allows for proposing the application of the OKS in those situations where the original version has been used, such as measuring HRQL improvement after knee replacement surgery [30] or studying surgery-related factors [57–59], but also to discriminate between patients in different clinical situations and to appreciate their evolution with time in view of its capacity to detect “moderate” improvements in patients.

## Conclusions

The Spanish adaptation of the OKS questionnaire is a valid tool for assessing the perception of HRQL of patients suffering from knee OA. It is well accepted by patients and shows psychometric properties that support its usefulness both for the assessment of a patient’s condition and its subsequent evolution. Its comparative utility is quite similar to that of tools that have been extensively used after their adaptation, like the WOMAC questionnaire. The incorporation of this type of tools in usual clinical practice will allow for appraising, in a valid and reliable way, the patient’s self-perception of HRQL as well as the outcome of health interventions addressed at them.

## Additional file

Additional file 1: Spanish-adapted version of the Oxford Knee Score - Spanish (Spain). (DOCX 23 kb)

## Abbreviations

BMI: Body Mass Index
CFA: Confirmatory Factor Analysis
CFI: Comparative Fit Index
CI95%: 95% Confidence Interval
EFA: Explanatory Factor Analysis
GDP: Gross Domestic Product
HRQL: Health-Related Quality of Life
ICC: Intraclass Correlation Coefficient
KMO test: Kaiser-Meyer-Olkin test
MCDI: Minimal Clinically Important Difference
MDC: Minimal Detectable Change
OA: osteoarthritis
OKS: Oxford Knee Score
RMSEA: Root Mean Square Error of Approximation
SEM: Standard Error of Measurement
TLI: Tucker-Lewis Index
VAS: Visual Analogue Scale
WOMAC: Western Ontario and McMaster Universities Osteoarthritis Index
