# Supplementary material for: Validation of the Spanish version of the Oxford knee score and assessment of its utility to characterize quality of life of patients suffering from knee osteoarthritis: a multicentric study
Source: Health Qual Life Outcomes. 2017 Sep 29;15:186. doi: 10.1186/s12955-017-0761-2 (PMC5622506; doi:10.1186/s12955-017-0761-2)
Supplement: Additional file 1: — Spanish-adapted version of the Oxford Knee Score - Spanish (Spain). (DOCX 23 kb) [file 12955_2017_761_MOESM1_ESM.docx]

**Additional file 1. Spanish-adapted version of the Oxford Knee Score - Spanish (Spain).
 © Oxford University Innovation Limited, 1998. All rights reserved.**

1. Durante las últimas 4 semanas…¿Cómo describiría el dolor que tiene normalmente debido a su rodilla?

| Ninguno | Muy leve | Leve | Moderado | Grave |
| --- | --- | --- | --- | --- |
|  |  |  |  |  |

1. Durante las últimas 4 semanas…¿Ha tenido algún problema para lavarse y secarse (todo el cuerpo) debido a su rodilla?

| Ningún problema | Muy pocos problemas | Problemas moderados | Dificultad extrema | Imposible hacerlo |
| --- | --- | --- | --- | --- |
|  |  |  |  |  |

1. Durante las últimas 4 semanas…¿Ha tenido algún problema para subir y bajar de un coche o para usar el transporte público debido a su rodilla? (sea cual sea el que suela utilizar)

| Ningún problema | Muy pocos problemas | Problemas moderados | Dificultad extrema | Imposible hacerlo |
| --- | --- | --- | --- | --- |
|  |  |  |  |  |

1. Durante las últimas 4 semanas…¿Durante cuánto tiempo ha podido andar antes de que el dolor debido a su rodilla se volviera grave (con o sin bastón)?

| Sin dolor/Más de 30 minutos | 16 a 30 minutos | 5 a 15 minutos | Solo por casa | Nada/dolor grave al caminar |
| --- | --- | --- | --- | --- |
|  |  |  |  |  |

1. Durante las últimas 4 semanas…Tras una comida (sentado/a a una mesa), ¿en qué medida le ha resultado doloroso levantarse de la silla debido a su rodilla?

| Nada doloroso | Ligeramente doloroso | Moderadamente doloroso | Muy doloroso | Insoportable |
| --- | --- | --- | --- | --- |
|  |  |  |  |  |

1. Durante las últimas 4 semanas…¿Ha estado cojeando al andar debido a su rodilla?

| Rara vez/ nunca | A veces, o solo al principio | Con frecuencia, no solo al principio | La mayoría del tiempo | Todo el tiempo |
| --- | --- | --- | --- | --- |
|  |  |  |  |  |

1. Durante las últimas 4 semanas…¿Podía arrodillarse y levantarse de nuevo después?

| Sí, fácilmente | Con poca dificultad | Con dificultad moderada | Con extrema dificultad | No, imposible |
| --- | --- | --- | --- | --- |
|  |  |  |  |  |

1. Durante las últimas 4 semanas…¿Le ha molestado el dolor debido a la rodilla en la cama por la noche?

| Ninguna noche | Solo 1 o 2 noches | Algunas noches | La mayoría de noches | Todas las noches |
| --- | --- | --- | --- | --- |
|  |  |  |  |  |

1. Durante las últimas 4 semanas…¿En qué medida ha interferido su dolor debido a la rodilla con su trabajo habitual (incluidas las tareas domésticas)?

| Nada | Un poco | Moderadamente | Mucho | Totalmente |
| --- | --- | --- | --- | --- |
|  |  |  |  |  |

1. Durante las últimas 4 semanas…¿Ha sentido que su rodilla podría no responderle o no soportar su peso repentinamente?

| Rara vez/ nunca | A veces, o solo al principio | Con frecuencia, no solo al principio | La mayoría del tiempo | Todo el tiempo |
| --- | --- | --- | --- | --- |
|  |  |  |  |  |

1. Durante las últimas 4 semanas…¿Podía hacer la compra para su casa usted solo/a?

| Sí, fácilmente | Con poca dificultad | Con dificultad moderada | Con extrema dificultad | No, imposible |
| --- | --- | --- | --- | --- |
|  |  |  |  |  |

1. Durante las últimas 4 semanas…¿Podía bajar un tramo de escaleras?

| Sí, fácilmente | Con poca dificultad | Con dificultad moderada | Con extrema dificultad | No, imposible |
| --- | --- | --- | --- | --- |
|  |  |  |  |  |

All licences to use the Oxford Knee Score in Spanish should be requested from Oxford University Innovation Ltd using the following link [https://innovation.ox.ac.uk/clinical-outcomes/patient-reported-outcome-measures/](https://correo.salud.madrid.org/owa/redir.aspx?C=8-a4shm3C0Gn58IP5Z4GC8rZVqv3WtRIPRdaxzSwLgGHXVvRcrvIe7dak_Bv9s88qfsQhp_jk1o.&URL=https%3a%2f%2finnovation.ox.ac.uk%2fclinical-outcomes%2fpatient-reported-outcome-measures%2f)"
